# A decreased number of circulating regulatory T cells is associated with adverse pregnancy outcomes in patients with systemic lupus erythematosus

**DOI:** 10.1002/iid3.731

**Published:** 2022-11-07

**Authors:** He‐Tong Li, Sheng‐Xiao Zhang, Jia‐Qi Zhang, Ting Cheng, Yan Liu, Hong‐Qi Liu, Min Hao, Jun‐Wei Chen

**Affiliations:** ^1^ Department of Obstetrics and Gynecology Second Hospital of Shanxi Medical University Taiyuan China; ^2^ Key Laboratory of Cellular Physiology, Ministry of Education Shanxi Medical University Taiyuan China; ^3^ Department of Rheumatology Second Hospital of Shanxi Medical University Taiyuan China; ^4^ Department of Information management Second Hospital of Shanxi Medical University Taiyuan China

**Keywords:** abortion, lupus, lymphocyte subpopulations, pregnancy, Treg cells

## Abstract

**Objective:**

As an autoimmune disease affecting women of reproductive age, systemic lupus erythematosus (SLE) is linked to adverse fetal and maternal outcomes. However, the status of peripheral lymphocytes in SLE patients with different pregnancy outcomes is unclear. This retrospective cross‐sectional study explored the relationship between lymphocyte subpopulations and pregnancy outcomes in married SLE female patients.

**Methods:**

The absolute numbers of peripheral T, helper T (Th)1, Th2, Th17, regulatory T (Treg), B, and natural killer (NK) cell subpopulations from 585 female SLE patients and 91 female healthy controls (HCs) were assessed. We compared the lymphocyte subpopulations in SLE patients with HCs and analyzed the absolute number and ratio of Treg cells according to pregnancy outcome in SLE patients.

**Results:**

SLE patients had decreased numbers of T, B, NK, Th1, Th2, Th17, and Treg cells and an imbalance in pro‐ and anti‐inflammatory cells (*p* < .05), as well as adverse pregnancy outcomes. In abortion patients, the number of Treg cells (*p* = .008) decreased, leading to an imbalance in effector T and Treg cells. The ratio of Treg cells was higher in SLE patients with nulliparity than in those with one or two parities.

**Conclusions:**

The absolute numbers of lymphocyte subpopulations in SLE patients decreased, which was associated with abortion and parity (*p* < .05). These results suggest that a loss of immune tolerance mediated by Tregs triggers pregnancy loss.

## Graphical abstract

Systemic lupus erythematosus (SLE) is a complex autoimmune disease that predominantly develops among women of childbearing age. Decreased number of regulatory T (Treg) cells and imbalanced Treg ratio was associated with adverse pregnancy outcomes in SLE. Normal Treg cell numbers and function is a prerequisite for healthy ongoing pregnancy.

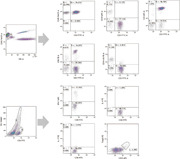

## 1 INTRODUCTION

Systemic lupus erythematosus (SLE) is a multiorgan and systemic autoimmune disease varying in severity across patients and over time. It is estimated that SLE affects about 1.5 to 11 per 100,000 persons worldwide.^1^ The incidence of SLE is six to sevenfold higher in females than males, predominantly developing in women of childbearing age.^2^ Pregnancy may induce SLE recurrence or exacerbation.^3^ Complications during pregnancy in the setting of SLE are more likely to occur and SLE is an important contributor to fetal and maternal mortality and morbidity. Obstetric complications plague pregnant SLE patients, mainly in terms of intrauterine growth restriction, preeclampsia, and preterm delivery.^4^, ^5^ Insight into the mechanism of pregnancy loss is needed to enable female SLE patients to achieve successful pregnancies.

Important immune alterations such as immune deficiency occur in SLE, and pregnancy is related to immune changes that ensure immune tolerance to the product of conception.^6^ The percentages of regulatory T (Treg) cells increase from the prenatal to the postpartum period are smaller in nulliparous women than parous women.^7^ The risk factors for poor pregnancy outcomes include antiphospholipid syndrome and lupus nephritis.^8^, ^9^, ^10^ However, alterations in the absolute numbers of Treg cells and other lymphocyte subsets in the peripheral blood of SLE patients and their impact on pregnancy loss are unclear.

A disturbance in the balance between effector T cells and Tregs contributes to the pathogenesis of SLE.^11^ However, the relationship between circulating Treg cells in SLE and pregnancy outcomes is unclear and data on the pregnancy outcomes of women with SLE are sparse. We evaluated circulating Tregs and effector T cells and explored the association of pregnancy outcome with SLE in married female patients.

## 2 METHODS

### 2.1 Participants

We consecutively enrolled 585 married female patients with SLE from the Department of Rheumatology at the Second Hospital of Shanxi Medical University from January 2014 to December 2019. The patients met the 1997 American College of Rheumatism classification criteria for SLE.^12^ We also enrolled 91 married female healthy controls (HCs) who visited the physical examination center or rheumatology clinic of the same hospital during the same period. In total, 676 peripheral blood samples were obtained at admission to compare the level of peripheral lymphocytes subpopulation between patients and HCs, including B cells, natural killer (NK) cell, T cells, and CD4^+^ T cell subsets. All participants' medical charts were reviewed for previous pregnancies. The participants ranged from 19 to 85 years of age. The exclusion criteria were malignancy; kidney, heart, or liver dysfunction; recent infection; and/or other autoimmune disease. Each SLE patient was treated after admission in accordance with EULAR 2019 recommendations for the management of SLE. The study was performed in accordance with all relevant tenets of the Declaration of Helsinki and was approved by the Second Hospital of Shanxi Medical University Ethics Committee. Written informed consent was obtained from each participant at the time of study enrollment.

Laboratory and demographic data were collected for all patients. Sterility was defined as the inability to conceive a child after having had intercourse for 1 year without contraception. The two assessed pregnancy outcomes were: (i) no abortion, defined as all pregnancies resulting in a live birth, and (ii) abortion, all abortions except induced abortions. Abortion included early pregnancy loss during the first trimester, late pregnancy loss (at 13−20 weeks of gestation), and intrauterine death (after 20 weeks of gestation).

### 2.2 Flow cytometry

To enumerate T cells, B cells, NK cells, CD4^+^ T cells, and CD8^+^ T cells, 50 μl of sample were placed in A and B TruCount tubes for immunofluorescence staining. CD8‐phycoerythrin (PE), CD4‐allophycocyanin (APC), CD3‐fluorescein isothiocyanate (FITC), and CD45‐peridinin‐chlorophyll protein (PerCP) antibodies were added to tube A. To tube B was added CD45‐PerCP, CD3‐FITC, CD16^+^56‐PE, and CD19‐APC antibodies. Stained cells were washed and incubated; 15,000 cells in the gate were assayed by flow cytometry (FACSCalibur; BD Biosciences).

**Figure 1:**
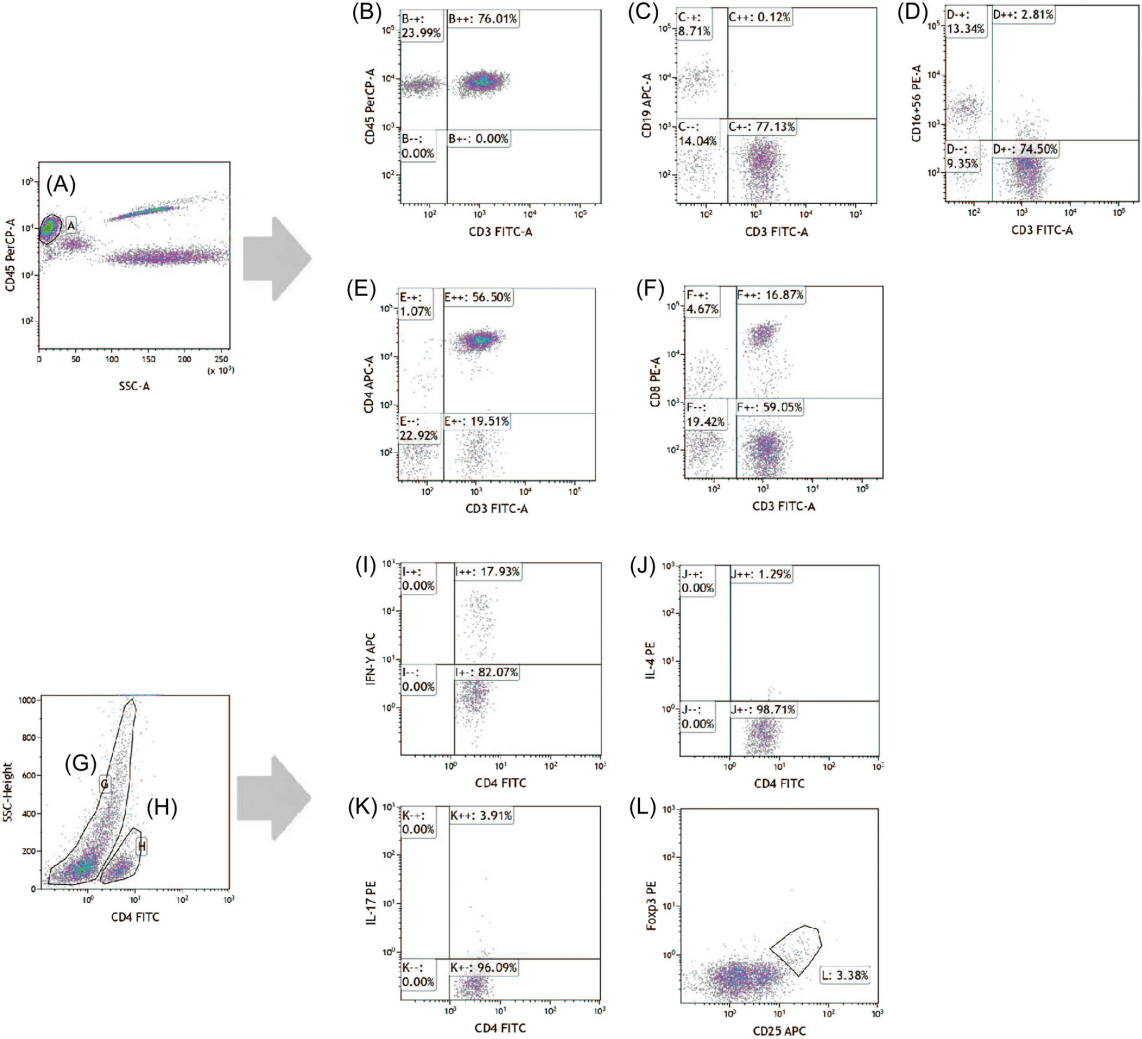
Analysis of peripheral lymphocytes and gating strategy. (A) Lymphocytes (CD45^+^). (B) T cells (CD45^+^CD3^+^). (C) B cells (CD45^+^CD3^−^CD19^+^). (D) NK cells (CD45^+^CD3^−^CD16^+^CD56^+^). (E) CD4^+^ T cells (CD45^+^CD3^+^CD4^+^). (F) CD8^+^ T cells (CD45^+^CD3^+^CD8^+^). (G) CD4^−^ T cells (CD45^+^CD3^+^CD4^−^). (H) CD4^+^ T cells (CD45^+^CD3^+^CD4^+^). (I) Th1 cells (CD45^+^CD3^+^CD4^+^IFNγ^+^). (J) Th2 cells (CD45^+^CD3^+^CD4^+^IL4^+^). (K) Th17 cells (CD45^+^CD3^+^CD4^+^IL17^+^). (L) Treg cells (CD45^+^CD3^+^CD4^+^CD25^+^Foxp3^+^). IFN, interferon; IL, interleukin; NK, natural killer.

To analyze helper T (Th)1, Th2, and Th17 cells, heparinized blood was stimulated with GolgiStop, ionomycin, and phorbol myristate acetate. Cells were transferred to tubes A and B labeled with anti‐CD4‐FITC. To tube A was added anti‐interferon‐γ‐APC and anti‐interleukin (IL)‐17‐PE, and to tube B, human anti‐IL4‐PE. When Treg cells were detected, anticoagulated blood was stained with anti‐CD25‐APC and anti‐CD4‐FITC. Cells were fixed and permeabilized for 30 min at 4°C in the dark, then stained with anti‐FOXP3‐PE; 10,000 cells in the gate were detected by flow cytometry. Cell Quest software was used to analyze CD4^+^ T‐cell subset frequencies. Absolute numbers of various CD4^+^ T subsets were calculated by multiplying the percentage of each CD4^+^ T subset by the total number of CD4^+^ T cells.^13^, ^14^, ^15^ The phenotypic characteristics of lymphocytes and CD4^+^ T cell subsets are shown in Figure 1.

### 2.3 Statistical analysis

Quantitative variables are expressed as means and standard deviations. Demographic parameters were compared by independent Student's *t*‐tests. Categorical variables were tested by the *χ* ^2^ test and Fisher's exact test. To compare pregnancy outcomes among the groups, a one‐way analysis of variance was used. A two‐tailed *p* < .05 denotes statistical significance. Statistical analysis was performed using SPSS v. 22.0 and GraphPad Prism v. 6.0 software.

## 3 RESULTS

### 3.1 Comparison of clinical data

Fetal and maternal health outcomes during pregnancy in SLE are a concern. A prototypical disorder of immune regulation is in SLE, we conducted this study to explore alterations in the absolute numbers of immune lymphocyte subsets in the peripheral blood as well as the relationship of adverse pregnancy outcomes. The baseline characteristics of the participants are shown in Table 1. The SLE patients were divided into sterility (*n* = 36), abortion (*n* = 213), and delivery with no abortion (*n* = 336) groups. Next, the 213 abortion SLE patients were subdivided into nulliparity (*n* = 31), one or two parities (*n* = 160), and multiparity (*n* = 22) groups. Pregnancy outcomes differed significantly between the SLE patients and HCs. Mean age and other demographic variables did not differ significantly.

**Table 1:** Baseline characteristics of the SLE patients and healthy controls^a^

	SLE	HCs	*t*/*χ* b	*p* b
Age (years)	42.81 ± 12.19	42.82 ± 14.42	0.006	.995
Gender	All female	All female	‐	‐
Marriage age (years)	23.68 ± 3.01	23.11 ± 3.66	−1.616	.107
Pregnancy outcome				
Sterility	36/585 (6.15%)	3/91 (3.30%)	20.728	<.001
Abortion history	213/585 (36.41%)	13/91 (0.17%)		
Delivery with no abortion	336/585 (57.44%)	75/91 (82.42%)		
Time of delivery				
0	31/213 (14.55%)	0/13 (0.00%)	3.463	.138
1 or 2	160/213 (75.12%)	10/13 (76.92%)		
≥3	22/213 (10.33%)	3/13 (23.08%)		
Number/ratio of lymphocyte subpopulation				
T (cells/μl)	883.33 ± 543.58	1336.78 ± 420.90	9.016	<.001
B (cells/μl)	167.05 ± 173.19	219.27 ± 96.11	4.12	<.001
CD4^+^ T (cells/μl)	405.94 ± 276.11	711.16 ± 234.43	11.114	<.001
CD8^+^ T (cells/μl)	441.18 ± 311.39	525.80 ± 192.78	3.455	.001
NK (cells/μl)	94.10 ± 79.78	294.86 ± 137.39	13.536	<.001
Th1 (cells/μl)	83.59 ± 93.97	115.95 ± 63.40	3.111	.002
Th2 (cells/μl)	6.23 ± 6.98	8.22 ± 12.45	2.033	.043
Th17 (cells/μl)	6.01 ± 6.36	7.60 ± 4.08	2.931	.004
Treg (cells/μl)	15.15 ± 11.13	30.65 ± 13.12	11.437	<.001
CD4^+^ T/CD8^+^ T (ratio)	1.10 ± 0.72	1.45 ± 0.48	4.413	<.001
Th1/Th2 (ratio)	19.24 ± 20.69	20.88 ± 23.22	0.662	.509
Th1/Treg (ratio)	7.02 ± 10.86	4.45 ± 3.76	−3.713	<.001
Th2/Treg (ratio)	0.41 ± 0.90	0.30 ± 0.42	−1.627	.105
Th17/Treg (ratio)	0.45 ± 1.05	0.27 ± 0.13	−3.242	<.001
T/Treg (ratio)	82.72 ± 99.88	58.10 ± 59.04	−2.249	.025
CD4^+^ T/Treg (ratio)	34.56 ± 34.60	29.74 ± 28.93	−1.222	.222
CD8^+^ T/Treg (ratio)	44.02 ± 62.39	23.79 ± 25.25	−4.74	<.001
B/Treg (ratio)	16.31 ± 24.45	9.29 ± 8.97	−4.346	<.001
NK/Treg (ratio)	9.43 ± 10.54	13.67 ± 18.92	2.829	.005

Abbreviations: HCs, healthy controls; NK, natural killer; SD, standard deviations; SLE, systemic lupus erythematosus; Th, helper T cells; Treg, regulatory T.

^a^ The values are means ± SDs for normally distributed variables, or *n* (%) for categorical variables.

^b^ *p* Values for differences between two groups by the independent‐samples *t*‐test for measurement data and *χ* ^2^ test and Fisher's exact test for categorical data.

### 3.2 Absolute number of lymphocytes and the Treg ratio

**Figure 2:**
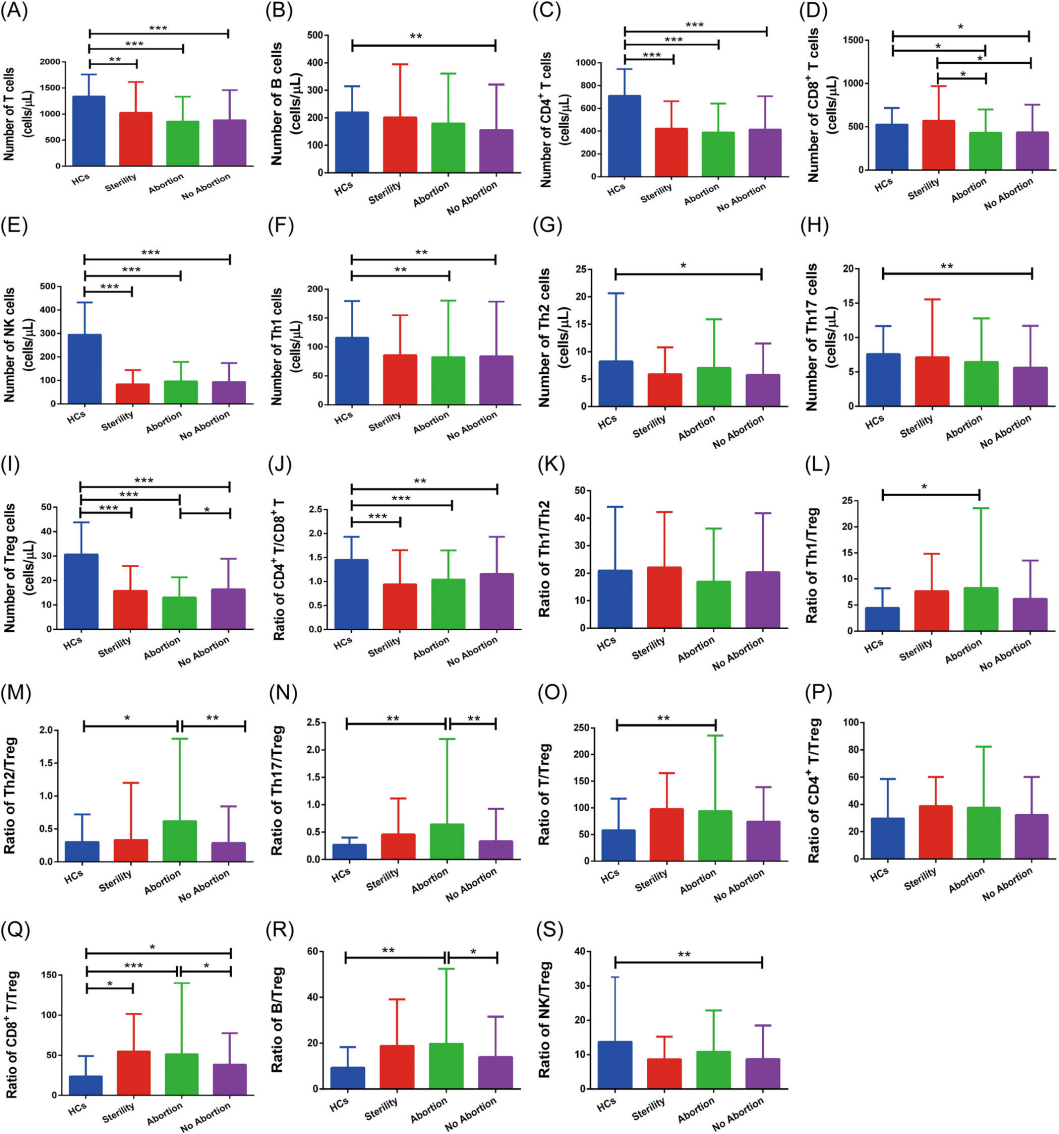
Relationship between peripheral lymphocyte subpopulations and pregnancy outcomes. Patients were divided into sterility (*n* = 36), abortion (*n* = 213), and delivery with no abortion (*n* = 336) groups. Statistical analysis was determined by one‐way ANOVA. (D) The CD8^+^ T cell numbers in the abortion and delivery with no abortion patients were significantly lower than in sterility patients. (I, M, N, Q, and R) The number of Treg cells, Th2/Treg ratio, Th17/Treg ratio, CD8^+^ T/Treg ratio, and B/Treg ratio were significantly lower in abortion patients than delivery with no abortion patients. (A−C, E−H, J−L, O−P, and S) No significant difference was found in the numbers of lymphocytes according to pregnancy outcome in SLE patients. ∗*p* < .05; ∗∗*p* < .01; ∗∗∗*p* < .001. ANOVA, analysis of variance; HCs, healthy controls; SLE, systemic lupus erythematosus; Th, helper T cells; Treg, regulatory T cells.

Compared with HCs, the SLE patients exhibited significant reductions in the numbers of T (*p* < .001), B (*p* < .001), CD4^+^ T (*p* < .001), CD8^+^ T (*p* = .001), NK (*p* < .001), Th1 (*p* = .002), Th2 (*p* = .043), Th17 (*p* = .004), and Treg cells (*p* < .001), implicating these cells in SLE pathogenesis. The SLE patients had higher Th1/Treg (*p* < .001), Th17/Treg (*p* = .001), T/Treg (*p* = .025), and B/Treg (*p* < .001) ratios and lower CD4^+^ T/CD8^+^ T (*p* < .001) and NK/Treg (*p* = .005) ratios than the HCs. The Th1/Th2 (*p* > .05) and Th2/Treg (*p* > .05) ratios did not differ significantly between the SLE patients and HCs (Figure 2).

### 3.3 Aberrant lymphocytes and adverse pregnancy outcomes in SLE

Aberrant lymphocytes are linked to adverse pregnancy outcomes in SLE. In abortion patients, the number of Treg cells (*p* = .010) was decreased compared to no abortion patients, leading to a significant imbalance in the CD8^+^ T/Treg (*p* = .044), Th2/Treg (*p* < .001), Th17/Treg (*p* = .004), and B/Treg (*p* = .023) ratios. Additionally, the CD8^+^ T cell number in abortion and no abortion patients was significantly lower than in sterility patients (*p* = .014 and .015, respectively). There was no significant difference in the numbers and ratios of other types of immune cells (Figure 2).

**Figure 3:**
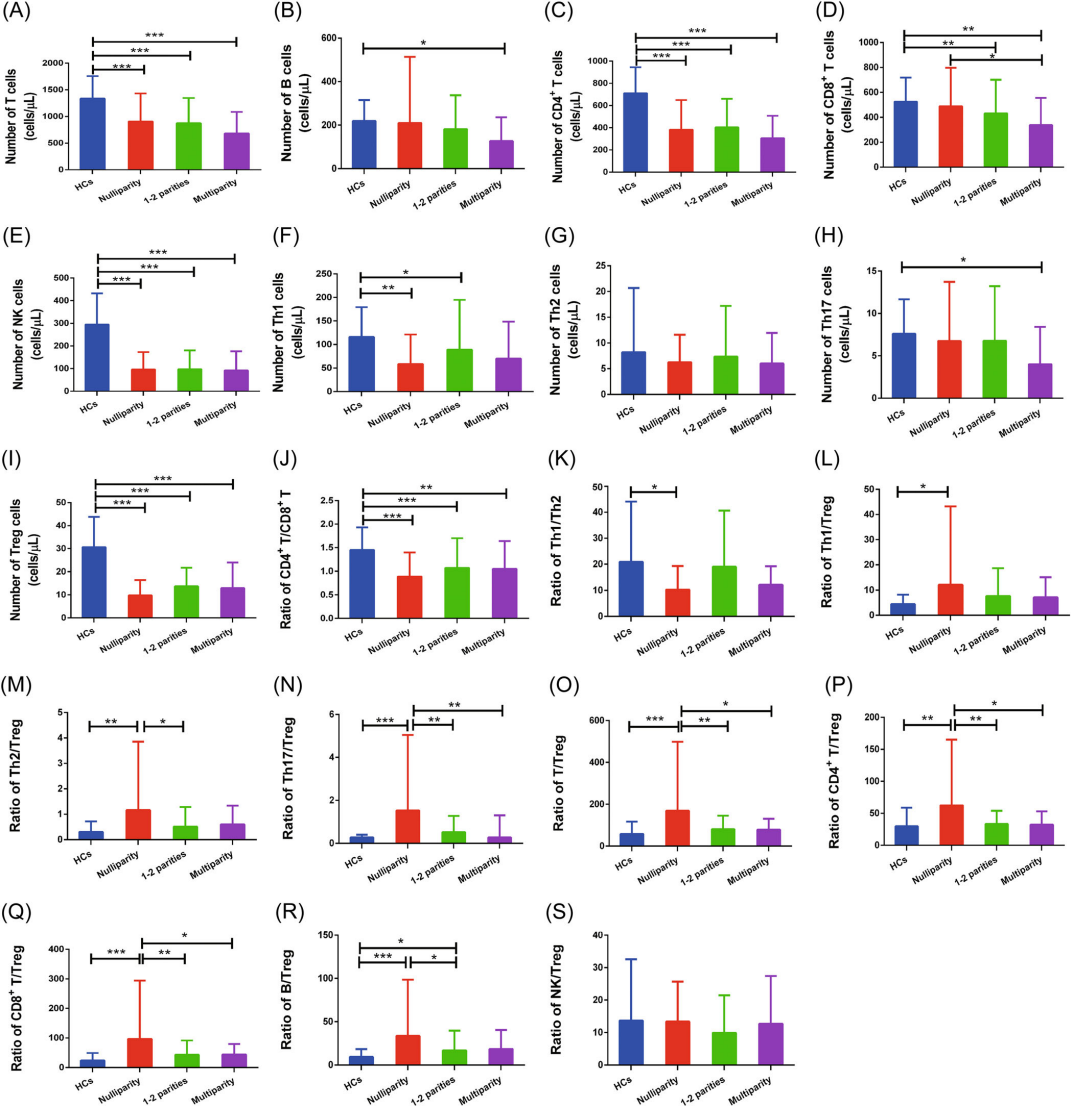
Peripheral lymphocyte subpopulations in the abortion subgroups. The abortion group patients (*n* = 213) were divided into nulliparity (*n* = 31), one or two parities (*n* = 160), and multiparity (*n* = 22) subgroups. Statistical analysis was determined by one‐way ANOVA. (A−C, E−L, and S) There was no difference in parity among the three groups. (D) The number of CD8^+^ T cells was higher in multiparous than nulliparous patients. (M, O, Q, and R) The Th2/Treg, T/Treg, CD8^+^ T/Treg, CD4^+^ T/Treg, Th17/Treg, and B/Treg ratios were higher in nulliparous patients than in those with one or two parities. (N and P) The T/Treg, Th17/Treg, CD8^+^ T/Treg, and CD4^+^ T/Treg ratios were significantly decreased in patients with one or two parities and multiparous patients compared with nulliparous patients. *p < .05; **p < .01; ***p < .001. ANOVA, analysis of variance; HCs, healthy controls; Th, helper T cells; Treg, regulatory T cells.

The T/Treg (*p* = .002), Th2/Treg (*p* = .010), Th17/Treg (*p* = .001), CD4^+^ T/Treg (*p* = .003), CD8^+^ T/Treg (*p* = .002), and B/Treg (*p* = .010) ratios were higher in nulliparous patients than in those with one or two parities. The T/Treg (*p* = .021), CD4^+^ T/Treg (*p* = .023), CD8^+^ T/Treg (*p* = .026), and Th17/Treg (*p* = .002) ratios were significantly lower in multiparous patients compared with nulliparous patients. Notably, the T/Treg, Th17/Treg, and CD4^+^ T/Treg ratios were negatively correlated with parity. There was no significant difference in the other lymphocyte parameters among the groups (Figure 3).

## 4 DISCUSSION

The numbers of immune cell subgroups were decreased in SLE patients, implicating this change in the pathogenesis of SLE. Immune cell subgroups orchestrate immune responses and promote immune tolerance to self‐antigens.^16^ Dysfunction of immune tolerance leads to the production of autoantibodies and the formation and deposition of immune complexes, leading to manifestations of SLE.^17^

The Treg, Th1, Th2, and Th17 cell subsets decreased in SLE patients, consistent with prior reports.^18^, ^19^ Treg cells produce immunomodulatory cytokines to suppress the inflammatory response and pathological damage.^20^ The decrease in Tregs in SLE patients is correlated with immunological metabolic abnormalities and tissue damage.^11^, ^21^, ^22^ A reduced number of T cells is associated with an increased risk of SLE.^23^ Similarly, an inadequate number of Tregs and downregulation of their activity impair the anti‐inflammatory environment and weaken immune tolerance against fetal rejection, thereby increasing the risk of spontaneous abortion.^24^, ^25^ Th1, Th2, and Th17 cells are regulated by Treg cells, and all participate in cell‐mediated immunity by secreting cytokines. Overstimulation of Th1 or Th2 immunity was reported in recurrent pregnancy loss.^26^ Also, the Th1–Th2 balance is broken in SLE. Th17 cells are proinflammatory and generally have the opposite effect of Treg cells.^27^ Pregnancy may be considered a conversion from proinflammatory and anti‐inflammatory conditions.^28^ Th17 and Tregs are in a state of dynamic equilibrium, which is disrupted in SLE.^29^, ^30^ The numbers of B cells and NK cells were reduced in SLE patients. B cells activate T cells by presenting self‐antigens and produce autoantibodies, which are key drivers of SLE.^31^ NK cells regulate homeostasis, secreting protective and proinflammatory cytokines to contribute to the exaggerated systemic inflammatory response to SLE.^32^, ^33^ Uterine NK cells not only have an immunoregulatory function but also regulate angiogenesis, trophoblast invasion, and spiral artery remodeling.^34^ A successful pregnancy and placental development depend on the function of secretory factors produced by maternal uterine immune cells within the decidua.^35^

The Th1/Treg, Th17/Treg, T/Treg, and B/Treg ratios were higher in SLE patients than HCs. The balance between adaptive immune cells and Treg cells is pivotal for immunological tolerance maintenance. The phenotypes and functions of maternal immune cells affect pregnancy outcomes. An increase in the Treg ratio is accompanied by a decrease in Tregs, modulating the immune response. Treg cells directly suppress the activation and proliferation of effector T cells and B cells.^23^ Autoantibody generation by clonal B cells against nuclear fragments is a feature of SLE.^36^ Treg cells also influence the progression of inflammation and immunity. Thus, a reduction in Treg cells causing a loss of tolerance may result in SLE.^37^ The dynamic changes in Th17 cells and Tregs are involved in the immune response.^38^ An absolute reduction in circulating Treg cells may lead to an imbalance between effector T cells and Tregs. Overall, in SLE, the absolute numbers of circulating Treg cells are decreased resulting in a loss of immune tolerance and increasing the production of a variety of autoantibodies, including against nuclear antigen.^39^

An absolute decrease in circulating Treg cells was correlated with pregnancy outcomes. The number of Treg cells significantly decreased and the Treg ratio increased in abortion patients compared to no abortion patients. Treg cells levels in the decidua were elevated compared to peripheral blood, implicating Treg cells in placental invasion.^40^ Successful pregnancy requires development of a placenta. Pregnancy involves adaptation to the semiallogeneic fetus, which is mediated by Treg cells. Nevertheless, the number of Treg cells was reduced in the peripheral circulation and partial decidua of women who had suffered a miscarriage. Murine spontaneous abortion is associated with a decreased frequency of Tregs, as in humans.^41^ Pregnancy with fetal loss is significantly associated with active disease at conception.^42^ The association of pregnancy with SLE represents a dilemma in which, during a normal pregnancy, Treg cells are augmented, whereas diminished numbers of and functionally defective Treg cells are associated with SLE.^43^ Systemic expansion of Treg cells occurs at very early stages in pregnancy; the cells accumulate at the fetus−mother interface, preferentially in the maternal decidua.^44^ Treg cells induce a tolerant microenvironment by interacting with other immune cells (e.g., NK cells) at the fetus−mother interface. The Treg cell number and ratio were not significant in sterility patients. SLE patients have normal fertility and sterility rates.^45^ SLE does not cause infertility unless the patient has been treated with cyclophosphamide, which can cause premature ovarian failure.^46^ Women with SLE have a higher rate of miscarriage and fewer live births.^47^ The immunopathological and clinical features of SLE during pregnancy also include the dysregulation of neutrophils and B cells.^38^

Transforming growth factor‐beta 1 (TGF‐β1) is a costimulatory factor of Treg cells that regulates their differentiation, suppresses their proliferation, and promotes apoptosis.^48^ A low TGF‐β1 level may downregulate Treg cells and promote vascular dysfunction, both systemically and in the placenta.^6^ Pregnant SLE patients could have placental pathology, emphasizing the necessity of immune cells during placenta formation. Dysregulation of the TGF‐β1/Treg cell axis impairs maternal tolerance to pregnancy. The blood levels of hormones increase during pregnancy, which may contribute to SLE.^49^ Females may be more susceptible than males to SLE in part because estrogen suppresses Tregs.^50^ The Treg ratio was associated with nulliparity, which is linked to a reduced percentage of Treg cells.^7^ Consequently, a decrease in circulating Treg cells and disrupted balance between Treg and immune cells may trigger fetal loss. Thus, obstetric and rheumatologic teams should work together, and antenatal follow‐up should include an ultrasound assessment of placental function and monitoring of peripheral blood Treg cell numbers. Treg‐based immunotherapy could improve pregnancy‐related outcomes.

It seems reasonable that boosting the number and activity of Treg cells should confer stronger immune tolerance.^51^ Since cytokines IL‐2 and TGF‐β are essential for the generation, function, and survival of Tregs. Low‐dose IL‐2 therapy can selectively target and expand the Treg population in SLE patients.^52^ Mesenchymal stem cell therapy for SLE has gained increasing attention, which could upregulate Treg proliferation through TGF‐β.^53^, ^54^ The possibility to expand and induce Tregs in vivo has recently also been considered through nanoparticles.^55^ Treg‐based therapeutic strategy has been successful in preclinical studies and has reached the clinic.

This study had several limitations. First, it used blood samples not obtained during pregnancy. Second, the marital and fertility histories were retrospective self‐reported data, which may result in recall bias. Third, the correlation of pregnancy with SLE disease activity and lymphocyte imbalances have not been fully elucidated. We could not control the changes in immune function during pregnancy in SLE patients. Large, prospective cohort studies focusing on the gestation and postpartum periods are needed to explore the roles of Treg cells in pregnant SLE patients.

## 5 CONCLUSIONS

In summary, a decreased number of Treg cells and an imbalanced Treg ratio were associated with adverse pregnancy outcomes in SLE. Decreased Treg cells is a significant risk factor for SLE, and may contribute to pregnancy loss. Immunological function is important for the pregnancy outcome of female SLE patients, and a sufficient number of functional Treg cells is a prerequisite for a healthy pregnancy.

## AUTHOR CONTRIBUTIONS

*Study design and manuscript writing*: He‐Tong Li and Sheng‐Xiao Zhang. *Data extraction and analysis*: He‐Tong Li, Sheng‐Xiao Zhang, Jia‐Qi Zhang, Yan Liu, and Hong‐Qi Liu. *Revised the manuscript*: Ting Cheng, Min Hao, and Jun‐Wei Chen. All authors approved the final version to be published.

## CONFLICT OF INTEREST

The authors declare no conflict of interest.

## Data Availability

All data generated or analyzed during this study are included.
